# Quantitative Assessment of the Risk of Introduction of Brucellosis From Ethiopia Into Germany Through the Importation of Small Ruminants

**DOI:** 10.1155/vmi/8036981

**Published:** 2025-06-13

**Authors:** Fekadu Gutema Wegi

**Affiliations:** Animal Health Research Program, Ethiopian Institute of Agricultural Research, P.O. Box 31, Holeta, Ethiopia

**Keywords:** brucellosis, Ethiopia, Germany, importation, risk assessment, sensitivity

## Abstract

**Background:** Despite the significant contribution of small ruminants to the improvement of societal livelihood, several factors hamper their production and productivity, chief among which are various production and reproductive diseases. Brucellosis is one of such diseases that causes huge economic loss and imposes trade restrictions.

**Methods:** A quantitative risk assessment was conducted from July 2023 to January 2024 to evaluate the risk of introduction of brucellosis into Germany via the importation of sheep and goat from Ethiopia. The QRA methods was applied by breaking it into different components, namely, hazard identification and characterization; developing a scenario tree; gathering scientific evidence about the probability of occurrence of these events from published sources; generating mathematical equations taking into account the reliability and variability of the evidences; and, finally, calculating the overall risk of the hazard introduction by running Monte Carlo simulation at 10,000 iterations using @ RISK software, Palisade Co.

**Result:** The overall probability of introducing brucellosis through the annual importation of sheep and goats from Ethiopia is 1.276 × 10^−7^ (fifth percentile = 3.07 × 10^−7^; 95th percentile = 3.08 × 10^−7^). The results of the sensitivity analysis using the tornado graph showed that the estimate's precision can be improved by 49%, 44%, and 35%, respectively, if the factors that contributed most to the uncertainty were changed by one standard deviation.

**Discussion and Conclusion:** If the animals (sheep and goat) pass through all mitigations as outlined in the study, the risk of brucellosis introduction into Germany through the importation of small ruminants from Ethiopia is generally low. The uncertainty around the risk estimate could be reduced if more animal-level prevalence data could be obtained and by employing more sensitive diagnostic tests such as ELISA to detect subclinically infected animals. It is recommended that animal health regulators of the two nations work closely to enhance disease diagnosis and surveillance capabilities.

## 1. Introduction

Ethiopia has a large population of small ruminants (sheep and goats), which are mostly kept in traditional subsistence production systems. According to the [1] report, Ethiopia owns 42.9 million sheep and 52.5 million goats in the world. This accounts for around 10% of Africa's and 4% of the world's small ruminant population [2]. In Ethiopia, small ruminant ownership ranges from 11% to 60% of households in the highland mixed agriculture regions and 41% to 95% in lowland pastoral regions of the country [3]. This shows that the livelihood of a large proportion of farmers depends on them due to their short generation intervals and high frequency of multiple births [4]. Small ruminants' production is important to alleviate the problems of malnutrition through the provision of meat and milk, and it also generates income and creates job opportunities. Research indicates that hides and skins are a major source of foreign exchange earnings, next to coffee in Ethiopia [5]. In 2020, Ethiopia earned 66.6 million US$, with the share of 64.20 and 2.4 million US$ from the export of small ruminant meat and live animals [6].

Despite this significant contribution of small ruminants to the enhancement of societal livelihood and boosting the national economy, several factors hamper their production and productivity, among which diseases are the major ones. Among the diverse range of diseases that affect the sheep and goat productivity, brucellosis is the major one affecting their reproductive performance and growth efficiency through late-term abortions, retention of placenta, infertility, and reduced milk production [7].

Brucellosis is the most important disease that causes huge economic loss to the small ruminant industry and causes significant public health impact and trade restrictions [8]. According to the 2016 annual health report of the then Ethiopian Ministry of Livestock and Fisheries, the disease is widely distributed in the country and causes a serious health problem in the sheep and goat population. The primary cause of SRB is *B. melitensis* bacteria, which are generally spread from infected animals to healthy animals and humans by direct contact with placentas and birth fluids as well as through consumption of tainted feed and water [9]. In humans, the disease is often characterized by a chronic debilitating illness and manifests as undulant fever, arthritic, liver, and spleen complications. *B. melitensis* has been controlled in most industrialized countries of Europe.

In 2000, Germany was declared officially free of bovine, ovine, and caprine brucellosis [10]. However, it remains endemic and is associated with an extensive negative impact on the productivity of flocks in low- and middle-income nations, such as the Mediterranean region, the Middle East, Central Asia, Sub-Saharan Africa, and parts of Latin America [11]. Hence, Germany's trade law prohibits the importation of small ruminants that do not meet the national veterinary officer's fundamental animal health requirements to prevent the resurgence and occurrence of brucellosis in Germany and other EU territories [12]. This shows that sanitary and phytosanitary (SPS) standards are receiving increasing attention within the framework of international trade.

Even though each nation exists in an exclusively different situation in terms of animal disease prevalence, product quality, and safety, the World Trade Organization (WTO) encourages a free trade exchange among the nations. Accordingly, the main markets for Ethiopian live animal exports have been its neighbors, including Somalia, Sudan, Yemen, Egypt, and other Middle Eastern nations, according to the Ethiopian Revenue and Customs Authority 2018/19 report. Being part of Ethiopia's government plan to generate more income from small ruminants' trade, the newly proposed trade partners concerning these commodities (sheep and goat) are Europe, Asia, and South America. Due to recent structural adjustments in the beef and dairy sectors, the livestock population is declining in Europe from December 2012 to 2022 [13].

In Germany alone, the decline of the livestock population is equal to 33% of the EU's overall reduction, which makes it the net importer of sheep and goat meat to fulfill the consumer demand. However, despite substantial demand for this commodity, exports have become a challenge due to the stringent sanitary requirements and repeated bans. By understanding the supply potential of Ethiopia and the growing demand of the importing countries such as Germany, Ethiopia wants to penetrate into the EU market by fulfilling SPS and other quality requirements, rules, and regulations governing the market. Hence, the risk of introduction of pathogens into Germany, in association with imported commodities, shall be scientifically assessed using available evidence. Therefore, the objective of this quantitative risk assessment (QRA) was to evaluate the risk of introduction of brucellosis into Germany via the importation of subclinically infected goat and sheep from Ethiopia.

## 2. Materials and Methods

### 2.1. Study Area and Population

This QRA was conducted by using published data, outbreak reports, and the national surveillance report of small ruminant brucellosis from all over Ethiopia. The study populations are all sheep and goat ready for marketing.

### 2.2. Hazard Identification

The hazard of interest in this case is brucellosis, a bacterial disease that causes high morbidity in adult animals and significant loss of newborns, and therefore has a high economic and public health importance. *Brucella* is a gram-negative coccobacillus causing infection mainly in livestock and animals, and humans acquire the infection through contact with infected animals [14]. Infection with *B. melitensis* is one of the most important causes of abortion in goats and sheep.

### 2.3. Evidence Gathering and Parameter Estimation

The required numerical inputs for this QRA model, that is, data on country level, herd, and animal prevalence of brucellosis; volume of live animal exports; antemortem inspection efficacy; effectiveness of animal health surveillance; and diagnostic efficacy, were gathered from published sources through an electronic search engine, using the following key terms: “small ruminants,” “goat,” “sheep,” “Brucellosis,” and “Ethiopia.” Data from the Ethiopian MoA and expert opinion solicitation were additionally used to estimate the likelihood of pathogen entry, establishment, and spread into importing countries. Next, a set of questions about the identified hazard was created using EPOA, and eventually, a scenario tree was developed.

### 2.4. The QRA Methods

**Figure 1:**
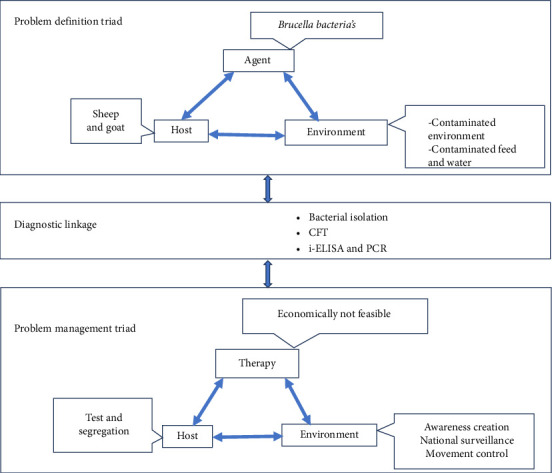
Epidemiologic problem-oriented approach (EPOA) methodology for identification of brucellosis in sheep and goat.

**Figure 2:**
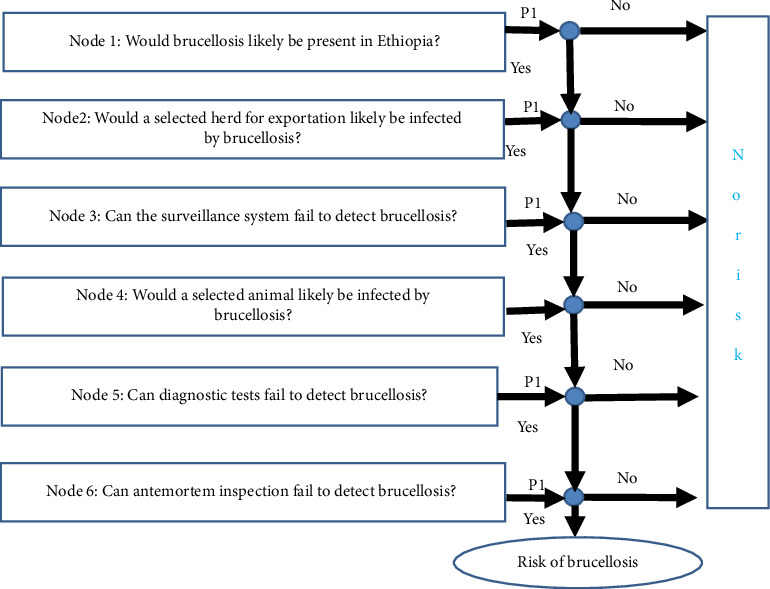
Scenario tree for brucellosis.

**Figure 3:**
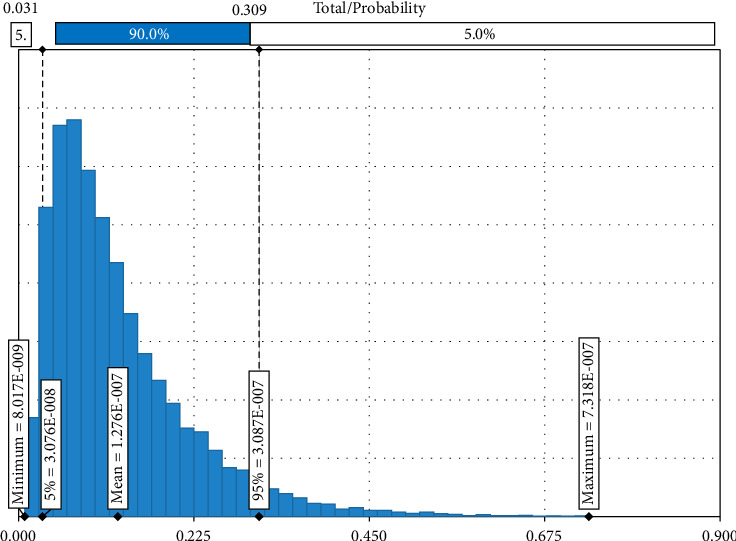
Overall probability of introducing brucellosis through shoat importation from Ethiopia.

The QRA method is a standard procedure that has been used extensively to assess the likelihood that an animal pathogen or disease could be introduced (enter) and establish and spread within a territory. Numerical data of small ruminant brucellosis for this QRA were obtained from outbreak reports, surveillance/monitoring activities, and various epidemiological studies conducted in Ethiopia. The data were analyzed following the QRA guidelines of the World Organization for Animal Health [15]. The QRA methodology was divided into four sections: (1) hazard identification (Figure 1), (2) development of a scenario tree (Figure 2), (3) evidence gathering and parameter estimation, and (4) developing mathematical equations, and finally calculating the likelihood of hazard introduction.

### 2.5. Developing a Scenario Tree

A method to obtain the discrete outcomes for the random variables is referred to as a scenario tree. It is a useful tool to structure and analyze information for decision-making concerning the prevention of animal diseases [16]. A path through the tree is called a scenario and consists of realizations of all random variables in all periods. Each node represents a possible realization of the stochastic process for a given stage and is associated with a probability. Connectors define the transition probability from the ancestor node to the successor node.

  Node 1: Would brucellosis likely be present in Ethiopia? According to numerous reports, brucellosis is prevalent in Ethiopia [17] and is responsible for more sickness, misery, and economic loss than any other zoonosis [18]. It is one of the renowned chronic diseases that can hinder the production performances of small ruminants through morbidity and reproductive losses [19]. Brucellosis is a common problem of sheep and goats in pastoral areas of Ethiopia such as Afar, Borana, and Omo valley and in the lowland of Tigray where there is a large population of goat and sheep. *B. melitensis* is the main cause of small ruminant brucellosis globally with occasional isolation of *B. abortus* from sheep and goats sharing the same environment with cattle.
  Therefore, *P*1 = …RiskUniform (0.03, 0.07).
  Node 2: Would a selected herd for exportation likely be infected by brucellosis? Brucellosis has been reported in small ruminants from different regions of Ethiopia It is a contagious bacterial disease which can get transmitted through direct contact with the infected animal, excreta, aborted fetus, amniotic fluid, placenta, and contaminated environment. A seroprevalence report of 13.7% by the authors in [20], 5.8% by the authors in [21], and 2.97% [22] depicts that small ruminant brucellosis is an endemic disease in Ethiopia. So, if one infected animal exists in the herd, there is a greater likelihood of this disease dissemination in the entire flock.
  Therefore, *P*2 = …RiskUnform (0.11, 0.28).
  Node 3: Can the surveillance system fail to detect brucellosis? Animal health surveillance refers to the collection, processing, and dissemination of animal health and disease information. Surveillance systems provide useful information for effective disease prevention and control, thereby improving food system productivity and food security, animal welfare, economic development, and access to international trade [23]. Hence, it is important to develop efficient, effective, and sustainable surveillance systems to detect emerging and exotic diseases promptly. The surveillance system will almost certainly receive positive reports of diseases of interest when identified. However, when the system does not receive any report of cases, it cannot distinguish between “no case detected” and “failure to report” [24]. However, a weak surveillance system inhibits the rapid detection of cases in countries with low income since they cannot afford comprehensive routine surveillance, hence limiting testing of all suspected cases [25].
  Therefore, *P*3 = …RiskUniform (0.3, 0.35).
  Node 4: Would a selected animal likely be infected by brucellosis? Brucellosis has been recognized in Ethiopia in 1970s [26] and since then, the disease has been noted as one of the important livestock diseases in the country [27–29].
  Therefore, *P*4 = …RiskUniform (0.03, 11%).
  Node 5: Can diagnostic tests fail to detect brucellosis? The diagnosis of *Brucella* infections can be made by culture, serological tests, and nucleic acid amplification assays [30]. The gold standard for diagnosis still is a bacterial culture [31]. However, diagnosis can be frequently delayed and often missed especially in the developing countries [31].
  Therefore, *P*5 = …RiskUniform (0.04, 0.11).
  Node 6: Can antemortem inspection fail to detect brucellosis? Brucellosis is a chronic bacterial disease which is manifested by late-term abortion, birth of weak offspring, reduced milk yield, and others. During the pregnancy stage, the production of erythritol sugar in the placenta supports multiplication of the *Brucella* bacteria and so abortion occurs. The detection of brucellosis by antemortem inspection is difficult in nonpregnant and male animals unless different diagnostic tests are employed. The difficultly in diagnosis may be due to the chronic nature of the diseases and the requirement for superior skill.
  Therefore, *P*6 = …RiskUniform (0.1, 02).

### 2.6. Developing a Mathematical Equation

After gathering scientific evidence, each node in a scenario tree (*P*1–*P*6) was given the appropriate numerical value (quantitative estimate). The estimates at each node were expressed as a range of values determined by the source documents or their natural domain (all possible values). Based on the ranges and estimates, RiskUniform distribution was used to capture variability or uncertainty around the risk estimate (Table 1) at each node.

### 2.7. Mathematical Calculations

The distribution of all possible outcomes of the hazard was generated by simulating the values at each node by running a Monte Carlo simulation with 10,000 iterations by using @ RISK software, Palisade Co. The sum of the product of the probability distributions, designated as *P*1–*P*6, was considered as the overall probability. The probability of a single animal being infected in the consignment was estimated using 1 − (1 − *P*)^*n*^, derived from the binomial law. The number of years until the first infected animal is exported was modeled using an exponential distribution (1/the mean number of infected animals exported per year). Finally, sensitivity analysis was performed to determine the extent to which a model's overall output is dependent on the uncertainty or variability in any one variable.

## 3. Result

### 3.1. Overall Probability

The Monte Carlo simulation revealed that there was an overall likelihood of 1.276 × 10^–7^ (fifth percentile = 3.07 × 10^−9^, 95th percentile = 3.08 × 10^−7^) of introducing brucellosis through the annual importation of sheep and goats from Ethiopia (Figure 3). Given that all the mitigation options are in place, the probability of a number of brucellosis-infected sheep and goat that could be imported into Germany is less than 31 animals in a million at 95%. There is only a 5% chance that the probability of brucellosis-infected sheep and goat that could be exported to Germany is greater than 31 in 10 million (Figure 3).

### 3.2. Sensitivity Analysis

**Figure 4:**
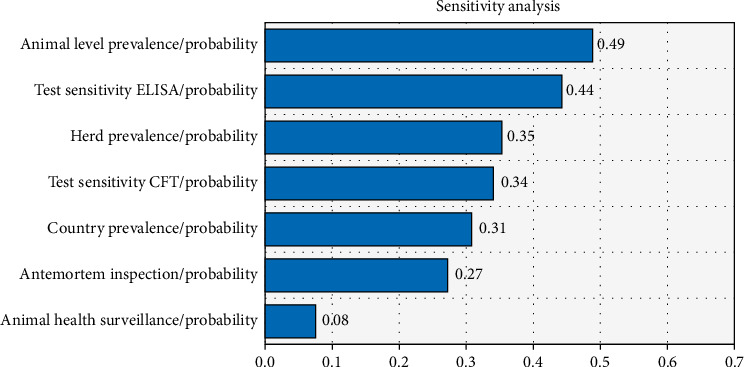
The variables contributing to the uncertainty of brucellosis infection risk estimates.

The results of the sensitivity analysis using the tornado graph showed that the factors that contributed most to the uncertainty of the overall probability of brucellosis introduction into Germany were the animal-level prevalence, test sensitivity (ELISA in this case), and herd prevalence (Figure 4). The estimate's precision can be improved by 49%, 44%, and 35%, respectively, if the factors that contributed most to the uncertainty were changed by one standard deviation.

## 4. Discussion

**Figure 5:**
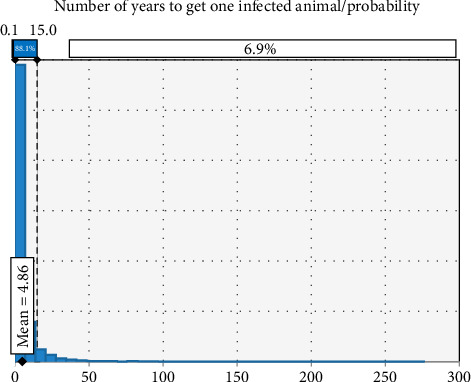
Graph showing the average number of years to get one infected animal.

In most of the developing world, brucellosis is an endemic zoonotic disease that causes the livestock industry to suffer greatly. This illness prevents access to foreign markets and limits economic growth [31]. In the present study, the baseline risk estimate derived from epidemiological data suggests that the risk of brucellosis spreading to Germany via the importation of sheep and goat from Ethiopia is very unlikely and also takes a long time to get one infected animal (Figure 5). This is because the probability of infected animals passing all existing disease screening and diagnosis techniques at the control point of both countries is very rare. Articles 229–239 of the EU regulate the import of live animals and their products from third countries to prevent the spread of disease. These articles stipulate that imports are only permitted from establishments and countries that meet all requirements regarding the health status of their territory and the ability to monitor and control endemic and emerging diseases [37]. Ethiopia is one of the countries with a large population of small ruminants, which also demands a reliable international market for this commodity. Therefore, much has been performed to be able to penetrate and maintain the level of their competency on the international market, to comply with standards set by importing countries, and to meet other demanded requirements. According to the sensitivity analysis using a tornado graph, the estimates that contributed most to the uncertainty surrounding the overall probability of brucellosis introduction into Germany were animal-level prevalence, test sensitivity (both ELISA and CFT), and herd prevalence. Given that the animals go through all mitigations in succession, the risk of brucellosis introduction into Germany through live animal importation is generally low. Furthermore, as per numerous research findings [38], brucellosis risk could be diminished by vaccination, early detection, and elimination of infected animals, restriction of animal movement, and enhancement of biosafety protocols.

## 5. Conclusion

According to the current QRA, the factors that contributed most to the uncertainty around the overall probability of brucellosis introduction into Germany were animal-level prevalence, test sensitivity (ELISA), and herd prevalence. Therefore, reducing the prevalence of the disease at the animal level, employing very sensitive diagnostic tests such as ELISA, and reducing the prevalence of the disease at the herd level are crucial to minimize the quantity of diseased animals that might be brought into Germany. Therefore, the MoA and other pertinent agencies ought to endeavor to reinforce measures for mitigating brucellosis-related ailments, such as enhanced methods for disease surveillance and inspection and implementation of immunization campaigns. In addition, there should be transparency and integration among the two nations' approaches in regard to live animal trading and brucellosis mitigation. Ethiopia will generally seek to increase quarantine and diagnostic services while resolving border porosity.

### 5.1. Limitation of the Study

Intraregional and cross-border trade of shoat (sheep and goat) is largely unrecorded and therefore the data on the small ruminant annual export volume of Ethiopia were not obtained accurately. So, the analysis was performed with the only available data. In addition, the current analysis was performed by using one type of distribution (RiskUniform), where a more probable estimate and higher certainty can be obtained if the other distributions such as the PERT distribution were used.

## Figures and Tables

**Table 1:** Variables, values, and distribution used for analysis of numerical data for QRA.

Variables	Min	ML	Max	Distribution	Probabilities	Reference
Country prevalence	0.03396		0.07356	RiskUniform	0.054975391	[32]
Herd prevalence	0.11026667		0.285225	RiskUniform	0.158465447	[33]
Animal-level prevalence	0.026625		0.108442857	RiskUniform	0.097863856	[21]
Surveillance failure	0.3		0.35	RiskUniform	0.303602955	[34]
Sensitivity of ELISA	0.0256067		0.088667	RiskUniform	0.025975007	[35]
Sensitivity of CFT	0.043		0.105857	RiskUniform	0.070132647	[36]
Antemortem inspection	0.1		0.2	RiskUniform	0.299159105	[35]
No. of animals exported/year (exp)	724,646	1,506,244	18,062,866	RiskPert	1802657.649	[34]
No. infect/exported/yr					*P* ∗ exp = 0.1734	
No. of yrs to get 1 infected				RiskExpon (1/*p* ∗ exp)	12.4224	

## Data Availability

The data that support the findings of this study are available from the corresponding author upon reasonable request.

## Nomenclature

CFT: Complement fixation test
ELISA: Enzyme-linked immunosorbent assay
EPOA: Epidemiologic problem-oriented approach
EU: European Union
MoA: Ministry of Agriculture
QRA: Quantitative risk assessment
SPS: Sanitary and phytosanitary
SRB: Small ruminant brucellosis
WTO: World Trade Organization
